# Evidence-Based Medicine in Iberoamerica: Problems and Possible Solutions

**DOI:** 10.1371/journal.pmed.0020277

**Published:** 2005-08-30

**Authors:** Zulma Ortiz, Pablo Perel, Jordi Pardo

**Affiliations:** **1**Argentine Collaborating Center of the Iberoamerican Cochrane NetworkBuenos AiresArgentina

We want to congratulate Chinnock and colleagues, who summarize very well the main problems that evidence-based medicine faces in developing countries [1]. As members of the Iberoamerican Cochrane Network, we would like to share some lessons learned and highlight possible solutions to the problems identified by Chinnock et al. [1].

We have learned from the experience of working in and with Latin American countries that one of the first barriers to overcome is inequity in accessing evidence. The second barrier is the English language. Efforts have been made by our network to overcome both barriers by providing free access to Biblioteca Cochrane Plus (BCP) (http://www.bibliotecacochrane.net). In addition to systematic reviews and protocols, this database contains evidence-based information not indexed in other sources.

However, ensuring access does not necessarily mean that reviews will be used in decision making. Many local problems do not appear in the BCP material, but those that are most prevalent and those with high impact on public health and clinical practice of these countries have been reviewed. Nevertheless, few health professionals apply the results of such reviews. One possible solution is that Cochrane centers, groups, or fields, most of which are based in developed countries, could invest resources in mass dissemination and promote their activities through organizations such as the Pan American Health Organization. This would encourage not only the use of systematic reviews, but also promote an interest in the Cochrane Collaboration from health authorities in the Americas.

Another aspect revealed in Chinnock et al.'s article is the need to get more people from developing countries involved in writing and peer-reviewing systematic reviews. The nature of the Cochrane Collaboration facilitates this, and we have had excellent results when working with several of its groups and fields. However, developing countries have a limited number of people qualified to participate in the writing and peer-reviewing of systematic reviews. Most of those who have the necessary skills also have an enormous load of teaching and clinical care, their salaries are insufficient to support a white-collar lifestyle, and, thus, private practice is the most common means of augmenting earnings. These economic issues are by far the major factor underlying the relative lack of research in developing countries [2]. Cochrane groups and centers based in developed countries should identify potential reviewers in developing countries and invest resources that provide them with spare time to devote to the promotion, production, and evaluation of systematic reviews. This idea is in line with the Millennium Development Goals [3], specifically number eight, which addresses the need to develop a global partnership for development. Nevertheless, the concerns exposed by Chinnock and colleagues in connection with the search for reviews performed in developing countries would decrease if the use of databases specific to these regions, such as LILACS (Literatura Latinoamericana en Ciencias de la Salud) in Latin America, was encouraged and if the use and development of these databases were supported.

Finally, we consider that advocacy on the importance of research and evidence-based public health should be strengthened in developing countries. This has been highlighted by Bernardo Houssay, the first Latin American honored with the Nobel Prize, who said, “Science is only science when it involves constant progress and improvement arising from research. Thus, there are only two possible standpoints: that of tuggers and that of others being tugged. In other words, you may either create knowledge at the same time others do, or accept a subordinate position and depend on what others produce.” When the response to his views was different from what he expected, he added, “It would not be ethical to base a research strategy on the unfair exploitation of sacrifices made by those with exceptional and determined minds. Wise countries do not live waiting for saints or miracles to occur” (quoted in [4]).
